# Observational Study of the Association between Air Cadmium Exposure and Prostate Cancer Aggressiveness at Diagnosis among a Nationwide Retrospective Cohort of 230,540 Patients in the United States

**DOI:** 10.3390/ijerph18168333

**Published:** 2021-08-06

**Authors:** Vishwaarth Vijayakumar, Michael R. Abern, Jyotsna S. Jagai, André Kajdacsy-Balla

**Affiliations:** 1 Department of Pathology, College of Medicine, University of Illinois at Chicago, 840 S Wood St, Suite 130 CSN, Chicago, IL 60612-4325, USA; ABalla@uic.edu; 2 Department of Urology, College of Medicine, University of Illinois at Chicago, 1801 W Taylor St #1e, Chicago, IL 60612-4795, USA; mabern1@uic.edu; 3 School of Public Health, University of Illinois at Chicago, 1603 W Taylor St, Chicago, IL 60612-4310, USA; jjagai2@uic.edu

**Keywords:** cadmium, air pollution, prostate cancer, heavy metals, cancer progression

## Abstract

Although studies have investigated cadmium and prostate cancer (PC) incidence and mortality, the role of cadmium in PC progression might be more clinically relevant. In this observational study, we assessed the association between air cadmium exposure and PC aggressiveness, with PC stage defined as metastatic or localized and Gleason grade defined as high (Gleason score ≥ 8) or low (Gleason score ≤ 6) among PC patients from the 2010–2014 US Surveillance, Epidemiology, and End Results database. The 2005 and 2011 National Air Toxics Assessment provided county-level air cadmium concentrations. Results were presented as odds ratios (OR) with 95% confidence intervals (CI) and were calculated using random intercept mixed effects logistic regression, comparing the 80th to 20th percentile of exposure. We adjusted for age, sociodemographic status, smoking prevalence, and overall air quality at the county level, and stratified by race, age, and degree of urbanization. The cohort consisted of 230,540 cases from 493 counties. Strong associations were observed in nonmetropolitan, urban areas: (OR 1.26, CI 1.14–1.39) for metastatic vs. localized and (OR 1.41, CI 1.27–1.57) for high- vs. low-grade PC where 40 million Americans reside. This study may be hypothesis-generating to inform future studies and public health measures.

## 1. Introduction

In 2018, there were an estimated 1,276,107 reported prostate cancer (PC) cases and 358,989 PC deaths worldwide [1]. Of these, an estimated 164,690 of cases and 29,430 of deaths were in the US [2]. Since it is well-known that PC progression can be slow, it is important to study environmental exposures that can accelerate carcinogenesis. Cadmium, in particular, was suspected to be associated with PC after reported cases among workers exposed to cadmium oxide in a nickel cadmium (Ni-Cd) battery factory in the UK [3]. A later study suggested that higher exposure to cadmium fumes was linked to PC mortality, although these results were not statistically significant [4]. Individuals exposed to high doses of cadmium through occupational exposure may die due to other complications such as lung cancer [5] and renal failure [6] before they develop PC. Therefore, it may be more informative to consider levels of exposure more relevant to the general population, and the relationship between cadmium and PC aggressiveness and progression. 

For nonsmokers, the largest source of cadmium intake comes from the diet. It is estimated that an average adult male consumes 0.35 µg of Cd/kg/day [7]. Since the average weight of an adult male in the US is approximately 89.8 kg, this means that the average adult male consumes approximately 31.4 µg of Cd daily through the diet. However, this estimate can vary depending on the population and methods used [8,9]. Factors that affect the amount of cadmium present in crops include use of phosphate fertilizers, nearby sources of contamination, soil pH, and cultivating practices [10]. Smoking is also a major route of cadmium exposure as smokers have been found to have more cadmium deposited in their organs than non-smokers [11]. In rural areas, the ambient air concentration of cadmium varies from 0.1 to 5.0 ng/m^3^, and in urban areas this value can vary from 2 ng/m^3^ to 15 ng/m^3^, but in industrial areas, these concentrations can be as high as 150 ng/m^3^ [12]. The Agency of Toxic Substances and Disease Registry (ATSDR) states that the amount of cadmium inhaled in the air is negligible except near cadmium-emitting facilities, which include smelters, mines, waste incinerators, coal and fossil fuel combustion plants, and various manufacturing facilities [12]. 

Assuming most individuals inhale approximately 10 cubic meters of air per day, the amount of cadmium absorbed through the air could be a substantial amount of daily cadmium intake near cadmium-emitting facilities. Up to 50% of inhaled cadmium can be absorbed directly into the blood [13] compared to a gastrointestinal absorption rate of approximately 5% for dietary cadmium [14]. In addition, ambient air cadmium can deposit in the soil, water, and in-house dust, leading to other routes of exposure [15].

Previous cohort studies have primarily focused on dietary cadmium intake and PC incidence and mortality, but have provided conflicting results [16,17]. However, initially, the focus was on cohorts that were occupationally exposed to cadmium by inhalation in factories. For this reason, air cadmium exposure might be a more relevant form of cadmium intake than dietary cadmium. Likewise, PC aggressiveness might be a better measure of outcome than either PC incidence or mortality, because (1) many PC cases are indolent and not clinically meaningful, and (2) PC is a slow-progressing disease which results in a low percentage of cases progressing to mortality. Therefore, we aimed to study the relationship between air cadmium exposure and PC stage and grade at diagnosis in an ecologic manner using a large representative population-based sample of PCs in the entire United States. The results of this study are hypothesis generating for future, more local studies at the state and county level that could help inform public health measures. 

## 2. Materials and Methods

### 2.1. Outcome Data

Prostate cancer outcome data were gathered from the 2010–2014 U.S. Surveillance, Epidemiology, and End Results (SEER) database [18]. During this time period, participants were retrieved by SEER from 11 states with which we conducted this retrospective cohort study. We only considered patient data at the time of diagnosis to exclude any potential sources of bias due to differences in treatment. PC stage at diagnosis was categorized as either metastatic or localized using tumor-node metastasis (TNM) staging. 

For the purposes of this study, PC aggressiveness was defined in the form of stage and grade. Aggressive PC was defined as either metastasis of PC or high-grade PC. Stage and Gleason grade at diagnosis are the strongest predictors of PC mortality. In cases from 2008 to 2014, PC had a 5-year survival rate of 98% for all stages combined. However, metastatic PC had a 5-year survival rate of 30% [19]. The Gleason tumor grade scoring system is used by pathologists to evaluate how much the cells in the cancerous tissue still resemble normal prostate tissue under the microscope. Although the total Gleason score can vary from 2 to 10, scores from 2 to 5 are rarely assigned. Therefore, a Gleason score of 6 is typically the lowest designated cancer grade. Total Gleason score correlates with increased probability of mortality. In a study of the Surveillance, Epidemiology, and End Results (SEER) database in the US, patients that had a Gleason score of 8 ≥ had an adjusted hazard ratio of 2.820 (1.554, 5.115) when compared to patients with a Gleason score of ≤ 6 for prostate cancer-specific mortality [20]. 

Gleason grade was defined as low for Gleason scores ≤ 6 and defined as high for Gleason scores >8. When comparing high-grade vs. low-grade cases, Gleason scores of 7 were excluded as they denote an intermediate score, and there is a large variability in risk among these intermediate scores. In fact, an intermediate score is hard to interpret among pathologists, and therefore were excluded to further prevent any additional sources of bias. SEER recorded the county of residence of these PC patients as well. The county of residence was used for linking exposure data.

### 2.2. Exposure Data

**Figure 1:**
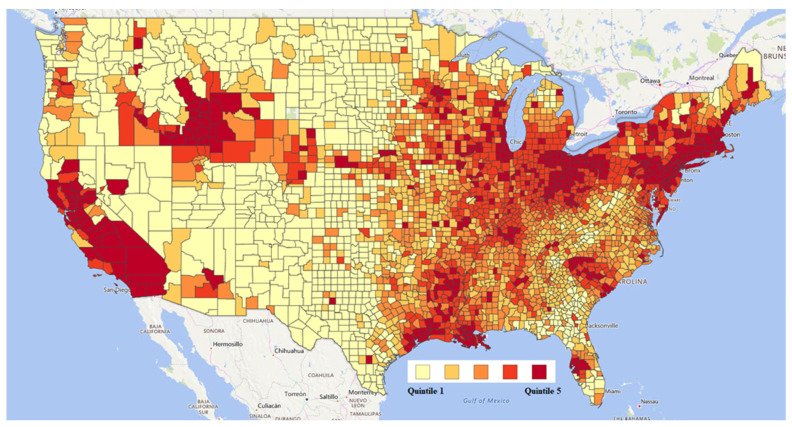
County-level air cadmium exposure concentration in the US by quintile.

Air cadmium exposure data were collected from the 2011 National Air Toxics Assessment (NATA) which was developed by the Environmental Protection Agency (EPA) to estimate concentrations of air toxics in the United States [21]. Since arsenic and lead are known causal agents of PC [22,23] and individuals that are exposed to cadmium in the air often are simultaneously exposed to arsenic and lead, we retrieved airborne lead and arsenic exposure data from NATA for comparison. At various point emission sources, cadmium, lead, and arsenic particulate matter were collected on high-quality filter paper as part of the 2011 National Emissions Inventory. Metals were dissolved in hot acid for extraction and were analyzed using inductively coupled plasma/mass spectrometry [24,25]. The total exposure concentration of cadmium compounds in the air was a county-level metric calculated by the EPA through models using weighted averages of air cadmium concentration levels measured at these various point emission sources. In this study, we utilized quintiles of cadmium exposure concentration for ease of interpretation which were similar to air cadmium levels reported by the ATSDR. QGIS open source software was used to construct a map of the US to display air cadmium exposure concentration by quintile at the county level to visualize regions in the US that have higher amounts of air cadmium than others (Figure 1). Lastly, we obtained air cadmium data from the 2005 NATA database as another time point for comparison with the 2011 database to assess the longitudinal accuracy and consistency in exposure measurements.

### 2.3. Covariates

We constructed a directed acyclic graph (DAG) to determine which factors to adjust for in our model. Indices for overall air quality and sociodemographic status at the county level were obtained from the EPA [26]. These indices represent two domains of five that are part of the Environmental Quality Index (EQI), which encapsulates many environmental factors into a single score. The air quality index assesses the concentrations of hazardous air contaminants. Likewise, the index for sociodemographic status considers education levels, socioeconomics, and reports of violent crime at the county level. Since the previous literature has demonstrated an increased risk of a PC case being metastatic compared to localized when considering poverty [27], and an association between terminal PC and air pollution [28], we included county-level EQI scores for sociodemographic status and air quality in our DAG to consider for adjustment. 

Demographic data such as county of residence, age at diagnosis, self-reported race and ethnicity were obtained from the SEER database for each patient. Since cadmium intake is usually a result of longitudinal exposure, we included age in our DAG. 

Smoking is a strong confounder of the amount of cadmium an individual is exposed to; however, smoking status is not provided in SEER data. Therefore, we included smoking prevalence at the county level in our DAG. Annually, the Centers for Disease Control and Prevention administers a survey called the Behavioral Risk Factor Surveillance System (BRFSS) through random telephone calls, in which it asks respondents whether they are current smokers. Smoking prevalence data from 2011 were used, which was represented as percentage of adults in each county who were current smokers. For this study, the BRFSS data were retrieved online from CountyHealthRankings.org (accessed on 8 August 2019) [29]. 

Air cadmium concentrations are included in the computation of the air domain index score. To address potential collinearity, we calculated models with and without the EQI air domain score for comparison. Since lead and arsenic are known carcinogens that are often present in the air with cadmium, these metals were considered covariates and were included in our DAG. According to our constructed DAG, we adjusted for age, county-level air quality score, score for sociodemographic status at the county level, and smoking prevalence at the county level. Since lead and arsenic are often present with cadmium and they are known carcinogens, we calculated separate analyses with cadmium, lead, and arsenic each as the exposure variable for purposes of comparison. 

The U.S. Department of Agriculture (USDA) classifies all counties in the US using nine Rural-Urban Continuum Codes (RUCC). We grouped the 9 RUCC codes into 4 categories which have been previously used for public health analyses: RUCC category 1 represented all metropolitan counties (original RUCC codes 1, 2, and 3). RUCC category 2 included counties with original RUCC codes 4 and 5 (nonmetropolitan counties with an urban population of 20,000 to 250,000). Original RUCC codes 6 and 7 (nonmetropolitan counties with an urban population of 2500–19,999) were grouped into RUCC category 3. Lastly, RUCC category 4 consisted of original RUCC codes of 8 and 9, which are completely rural counties with populations of less than 2500 [30]. Cases were stratified by their RUCC category code for further analysis. RUCC 1, 2, 3, and 4 used subsequently will refer to the category codes as defined above.

### 2.4. Statistical Analysis

The study cohort was first summarized by calculating descriptive statistics for race, PC aggressiveness, age, and county-level air cadmium, lead, and arsenic concentration within each RUCC type to see if there were any differences. For the study design, we calculated odds ratios (OR) and 95% confidence intervals (CI) assessing the relationship between county-level air cadmium exposure concentration and PC stage and grade at diagnosis using random intercept mixed effects logistic regression models where fixed effects were set at the state-level. Based on our DAG, analyses were adjusted for age at diagnosis, county-level sociodemographic index, county-level smoking prevalence, and county-level air quality index and were stratified by RUCC category. Cases were also stratified by race and age group within each RUCC category. OR included all cases but were calculated comparing the 80th vs. 20th percentile of cadmium exposure for ease of interpretation. We calculated similar 80th vs. 20th percentile OR for lead and arsenic for comparison as these metals are known carcinogens. All statistical analyses were performed using MATLAB (MathWorks, Natick, MA, USA), with *p* < 0.05 or 95% CI of OR not crossing 1.0 to be considered significant. Previous studies that that linked air cadmium to breast cancer informed our approach [31,32,33]. 

## 3. Results

### 3.1. Cohort Descriptives

The study cohort consisted of 230,540 PC cases from 493 counties reported by SEER, with most of the cases in RUCC 1 (~89%), followed by a roughly equal number of cases in RUCC 2 (~4%) and 3 (~6%), and the least number of cases in RUCC 4 (~1%). When comparing high vs. low Gleason grade risk, patients with a Gleason Score of 7, an intermediate score, were eliminated leaving 130, 317 cases for analysis (Table 1). White patients consisted of a majority (74%+) of the cases in all RUCC county types and were higher in more rural, less urban counties. Black patients consisted of approximately 16% of cases overall. Approximately 92% of all cases were localized (8% metastatic) and 30–36% of high Gleason Grade at diagnosis among all RUCC county subtypes. Most patients (~97%) were between the ages of 50 and 80, which was also fairly consistent between RUCC type. 

### 3.2. Exposure Data

Counties that tended to have higher air cadmium concentrations in 2011 also did so in 2005. In fact, 75% of the counties that were in the upper quintile of air cadmium concentrations in 2011, were in the upper 2 quintiles of air cadmium concentration in 2005. Likewise, 76% of the counties in the bottom quintile of air cadmium concentration in 2011 were in the bottom two quintiles of air cadmium of concentration in 2005. Lastly, ambient cadmium, lead and arsenic concentrations were higher and approximately equal in RUCC 1 and RUCC2 counties and lower in RUCC 3 and 4 counties (~1/2 the amount) (Table 1). These air cadmium concentrations were similar to past values recorded by the EPA in their NATA assessments.

### 3.3. Statistical Analyses

**Figure 2:**
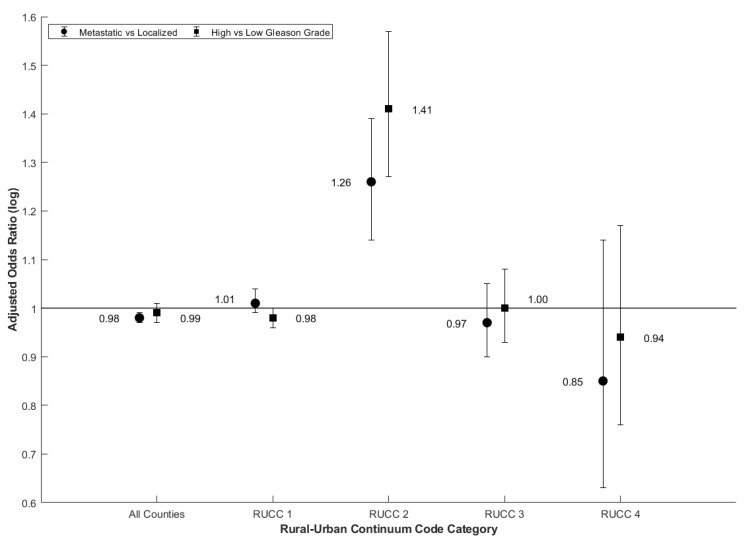
Adjusted odds ratios for metastatic vs. localized and high vs. low Gleason grade prostate cancer for the 80th vs. 20th percentile of cadmium exposure concentration (OR, 95% CI) from Surveillance Epidemiology, and End Results (SEER), a nationally representative cohort in the US. Note: Adjusted odds ratios were adjusted for age at diagnosis, county-level sociodemographic index, county-level smoking prevalence, and county-level air quality index.

Among all patients, higher levels of air cadmium exposure concentration were not associated with an increased likelihood of a PC case being metastatic (OR 0.98, CI 0.98–0.99) or having high Gleason grade at diagnosis (OR 0.99, CI 0.97–1.00) (Table 2 and Figure 2). Statistically significant associations were observed in RUCC category 2 counties: those in nonmetropolitan areas with urban populations of 20,000 to 250,000 (Table 2). These adjusted odds ratios for the 80th vs. 20th percentile of cadmium exposure were: (OR 1.26, CI 1.14–1.39) for metastatic vs. localized cases and (OR 1.41, CI 1.27–1.57) for high vs. low Gleason score cases, respectively (Figure 2).

The results did not differ with and without the air quality score in the model (results not presented). In addition, simply being in RUCC 2 does not explain a higher incidence of aggressive PC, since the percentage of patients that have aggressive PC is roughly the same in all 4 RUCC categories. For metastatic PC, this percentage varies from 7.4% to 8.9%, and for higher Gleason grade from 29.5% to 35.7% (Table 1). 

Generally, unadjusted and adjusted odds ratios were similar (Table 2). Adjusted odds ratios for cases comparing high vs. low Gleason grade were slightly larger for RUCC 2 and 4 for metastatic vs. not, but roughly equal for RUCC 1 and 3 (Table 2). However, only statistically significant odds ratios were found in RUCC 2 and were the largest compared to the other RUCC subtypes (Table 2). In RUCC 2, there is a trend that suggests that cadmium has a stronger association with metastatic PC and higher Gleason grade when compared to arsenic (metastatic vs. non-metastatic: 1.13, 1.08–1.18, high vs. low Gleason grade: 1.12, 1.06–1.18) and lead (metastatic vs. not: 1.00, 0.96–1.04, high vs. low Gleason grade: 1.04, 1.01–1.08; Table 2). Odds ratios among the three metals seem to be similar and smaller in the other RUCC county types (Table 2). For this reason, we focused our attention on studying RUCC category 2. 

**Figure 3:**
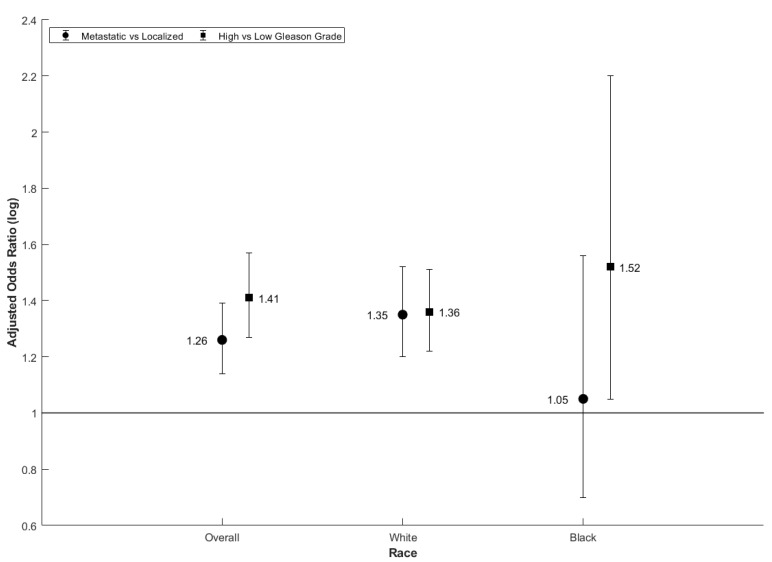
Adjusted odds ratios for metastatic vs. localized and high vs. low Gleason grade prostate cancer for the 80th vs. 20th percentile of cadmium exposure concentration (OR, 95% CI) by race in RUCC 2 counties. Note: Adjusted odds ratios were adjusted for age at diagnosis, county-level sociodemographic index, county-level smoking prevalence, and county-level air quality index.

In addition, to help understand the associations found in these counties, the cohort was stratified by race and age. For metastatic vs. localized cases stratified by race, odds ratios were only statistically significant among White patients in RUCC 1 (1.06, 1.03–1.08) and RUCC 2 (1.35, 1.2–1.52). For high vs. low Gleason Grade, significant odds ratios were observed among Black patients in RUCC 3 (1.28, 1.10–1.50), White patients in RUCC 2 (1.36, 1.22–1.51), and Black patients in RUCC 2 (1.52, 1.05–2.20). In these cases, odds ratios were higher for cadmium than they were for arsenic and lead (Table 3 and Figure 3).

When studying the cohort by age group, cadmium was associated with metastatic PC when compared to localized among all patients less than 60 years old (1.05, 1.02–1.08) and patients less than 60 years old in RUCC 1 (1.07, 1.03–1.12). For all patients in RUCC 2, this odds ratio was (1.26, 1.14–1.39) which was consistently found among all age groups. For all patients in RUCC 2 comparing high vs. low Gleason grade, the odds ratio for cadmium was 1.41 (1.27–1.57), which was also observed in all age subgroups. The odds ratio for patients in the age range of 61–70 in RUCC 3 was 1.11 (1.01–1.21). Again, odds ratios for cadmium were higher than for arsenic and lead in RUCC 2 (Table 4).

## 4. Discussion

The effect of cadmium on the aggressiveness of PC is of particular interest since PC has high incidence but has a relatively low rate of progression to more aggressive disease [1]. Among a large national cohort in the US, we found statistically significant associations between air cadmium exposure and high tumor grade and metastatic PC at diagnosis (Table 2) in RUCC 2 counties, with effects that differed somewhat by race (Table 3) and age (Table 4). In RUCC 2 counties, odds ratios were observed to be the largest among cadmium when compared to arsenic and lead (Table 2, Table 3 and Table 4). We were not able to identify any features of RUCC 2 counties that distinguished them from other RUCC counties, (Table 1) but this should be the goal of follow-up studies. Similar air cadmium exposure concentration measurements in counties between 2011 and 2005 NATA assessments indicate longitudinal accuracy and consistency in assessing air cadmium exposure. 

Previous literature studying the association between cadmium intake and PC aggressiveness fall into two categories: (1) mortality as endpoint and (2) biomarkers of aggressiveness at diagnosis as discussed below. 

When considering mortality (1), two meta-analyses showed no association between cadmium exposure and PC mortality in the general population [34,35]. However, mortality by itself may not accurately measure cadmium effect due to differences in treatment and the attenuation of the effect of cadmium, since most patients have an indolent form of PC. Only a few papers addressed signs of aggressiveness (2). Patients in Taiwan with higher serum and urinary cadmium levels had a significantly higher stage and Gleason grade [36]. Among a large Danish cohort, the incidence of aggressive and non-aggressive PC was not increased in men with high cadmium dietary exposure [16], but another large prospective study in Sweden discovered a rate ratio (RR) of 1.29 (CI: 1.08–1.53) for localized cases and RR of 1.05 (CI: 0.87–1.25) for advanced cases [17]. A population-based study in the US showed a tendency for higher incidence of aggressive PC in men when comparing the highest to lowest quartile, but this result was not statistically significant [37].

Unlike past studies of dietary cadmium that were interested in comparing the incidence of PC of varying aggressiveness separately [16,17,37], we calculated the odds ratio comparing the probability of aggressive to nonaggressive PC to understand the role cadmium may play in PC progression. In addition, it is often difficult to separate high dietary cadmium intake (heavily dependent on the ingestion of bread and potatoes) from adiposity, which is a well-known risk factor for PC aggressiveness and mortality [38]. Lastly, since the absorption of air cadmium is higher than that of dietary cadmium [14], our results may provide a more comprehensive assessment of environmental exposure to cadmium and support previous findings that PC patients with higher cadmium levels have a higher stage and Gleason grade [37]. 

Since we consistently found statistically significant associations in RUCC 2 counties, and the ATSDR indicated that air exposure to cadmium is only substantial near cadmium-emitting facilities, our hypothesis is that many cadmium-emitting facilities primarily tend to be in RUCC 2 counties, where approximately 40 million Americans live [30] with a substantial portion potentially subject to high cadmium exposure. The US Census Bureau obtains data regarding industry size at the county level, which are available publicly at the Data USA website [39]. After examining the industries in the upper two quintiles of air cadmium concentration in RUCC 2 counties, some putative sources include smelters, mining and quarrying, waste incinerators, coal and fossil fuel power plants, factories for manufacturing, and nickel–cadmium batteries. Future studies could identify more specific sources of high air cadmium exposure in RUCC 2 (and potentially other RUCC type) counties to develop public health strategies to curb cadmium emissions.

A link between Cd and PC was first suspected when workers in Ni-Cd battery factories that inhaled cadmium fumes had a higher risk of PC. In environmental and occupational exposures, air remains the main pathway of absorption. After absorption, Cd binds to metallothionein and is transported to the kidney. It is then filtered by the glomeruli and in the proximal tubules, the protein is degraded. A portion of the absorbed Cd is then transported to the rest of the body while the rest is slowly excreted. It is hypothesized that Cd reaches the prostate by mimicking certain hormones, but more studies are required [40]. Studies have found a correlation between air cadmium and blood cadmium and DNA damage [41,42], further suggesting the importance of air cadmium. Most of the literature addresses the biological effect of cadmium on the initiation and promotion of PC, with little attention to progression (an increase in genomic instability, tumor growth and metastasis) of already established malignancy. Most of the mechanism-driven studies in the literature may not be relevant as they utilize high, micromolar, in vitro concentrations of free, unbound cadmium salts which rarely, if ever reach the prostate. Cadmium overburden affects several cellular functions and organelles [43,44]. Of particular interest may be cadmium-induced depletion of antioxidant reserve in cells, with a decrease in intracellular levels of glutathione peroxidase and superoxide dismutase, leading to activation of redox-sensitive transcription factors such as NF-ƘB [45,46]. The few articles that do focus on low, nanomolar cadmium concentrations suggest that cadmium may act as a hormone disruptive agent and activator of signal transduction pathways that promote cell growth [47,48], but other possible mechanism(s) such as deregulation of cell proliferation, disturbance of tumor suppression functions, induction of oxidative stress, disturbance of DNA repair processes, and alterations of DNA methylation [49,50,51] may also contribute. 

The main limitation of our study, in common with most epidemiological studies, is its observational nature. In this study, using county-level cadmium exposure concentrations, it is impossible to assess a specific individual’s exposure to air cadmium and its absorption. In addition, cadmium exposure concentration could vary throughout the county as well, which means that different individuals living in the same county could be exposed to different amounts of air cadmium. Previous epidemiological studies have shown it is extremely challenging to estimate an individual’s amount of cadmium intake, absorption, and retention in the prostate. The SEER data are limited in that they only contained patient data from 11 out of the 40 states, have incomplete data about an individual’s location of residence and other health information, and take years to compile data which might make them less recent. Another limitation is the lack of information on frequency of PSA screening prior to diagnosis, which is associated with less aggressive prostate cancer and could be a confounding factor [52]. Therefore, it is possible that our calculated odds ratios are affected by other factors in addition to the presence of cadmium that are not accounted for in our model, such as body mass index (BMI), waist-to-hip ratio, comorbidity measures, and physical activity, which are not available in the SEER database. Lastly, it is challenging to completely adjust for possible coexposure with As and Pb when we are only interested in Cd exposure.

For these reasons, we consider this study to be hypothesis-generating to motivate local studies using state and local registries. The EPA publishes air toxic concentration data for census blocks and tracts, which are smaller than counties and might provide more precise information about an individual’s exposure and the link between the distance of a patient’s place of residence to a cadmium-emitting source and PC aggressiveness. Therefore, it is important to interpret these results with a balance between statistical findings and limitations of the study.

## 5. Conclusions

Instead of addressing the effect of environmental cadmium on PC incidence and mortality, we studied cadmium and PC aggressiveness to ignore clinically inconsequential cases of prostate cancer. We focused on air cadmium since this was initially suspected as the cause of PC incidence among workers occupationally exposed to high amounts in industrial scenarios. We found statistically significant associations in RUCC 2 counties (nonmetropolitan counties with an urban population of 20,000 to 250,000) where 40 million Americans reside, which suggests air cadmium is likely to be a risk factor for more aggressive cancer. Nevertheless, this study aims to be hypothesis-generating to motivate further studies to better investigate the effect of this form of cadmium on PC aggressiveness at a local level. Similar analyses could be undertaken to investigate other environmental factors and PC aggressiveness. Better understanding of the possible role of cadmium in PC progression could lead to preventive public health measures and therapeutic interventions.

## Figures and Tables

**Table 1:** Cohort descriptives of prostate cancer cases obtained from the 2010–2014 Surveillance, Epidemiology, and End Results (SEER).

Cohort Descriptors	No. of Cases (%) within Cohorts of 2 Measures of Prostate Cancer Aggressiveness									
	Metastatic vs. Localized Cohort *n* = 230,540 (493 Counties)					High vs. Low Gleason Grade Cohort ^a^ *n* = 130,317 (493 Counties)				
	All RUCC *n* = 230,540 (493 Counties)	RUCC 1 *n* = 205,302 (209 Counties)	RUCC 2 *n* = 9783 (47 Counties)	RUCC 3 *n* = 12,898 (172 Counties)	RUCC 4 *n* = 2557 (65 Counties)	All RUCC *n* = 130,317 (493 Counties)	RUCC 1 *n* = 115,986 (209 Counties)	RUCC 2 *n* = 5538 (47 Counties)	RUCC 3 *n* = 7358 (172 Counties)	RUCC 4 *n* = 1435 (65 Counties)
**Race**										
White	174,182 (75.5)	152,875 (74.5)	7931 (81.1)	10,970 (85.1)	2406 (94.1)	98,726 (75.8)	86,592 (74.7)	4486 (81.0)	6303 (85.7)	1345 (93.7)
Black	36,802 (16.0)	33,742 (16.4)	1204 (12.3)	1733 (13.4)	123 (4.8)	19,714 (15.1)	18,017 (15.5)	691 (12.5)	936 (12.7)	70 (4.9)
Other	11,767 (5.1)	11,213 (5.5)	469 (4.8)	79 (0.6)	6 (0.2)	6923 (5.3)	6628 (5.7)	249 (4.5)	41 (0.6)	5 (0.4)
Unknown	7789 (3.4)	7472 (3.6)	179 (1.8)	116 (0.9)	22 (0.9)	4954 (3.8)	4749 (4.1)	112 (2.0)	78 (1.0)	15 (1.0)
**Tumor Aggressive** **Type**										
Aggressive	17,318 (7.5)	15,194 (7.4)	869 (8.9)	1050 (8.1)	205 (8.0)	39,112 (30.0)	34,230 (29.5)	1900 (34.3)	2470 (33.6)	512 (35.7)
Non-Aggressive	213,222 (92.5)	190,108 (92.6)	8914 (91.1)	11,848 (91.9)	2352 (92.0)	91,205 (70.0)	81,756 (70.5)	3638 (65.7)	4888 (66.4)	923 (64.3)
**Age**										
≤49	6611 (2.9)	6133 (3.0)	193 (2.0)	245 (1.9)	40 (1.6)	4159 (3.2)	3854 (3.3)	122 (2.2)	156 (2.1)	27 (1.9)
50–59	49,347 (21.4)	44,572 (21.7)	1922 (19.6)	2416 (18.7)	437 (17.1)	29,167 (22.4)	26,313 (22.7)	1155 (20.9)	1445 (19.7)	254 (17.7)
60–69	97,255 (42.2)	86,606 (42.2)	4163 (42.6)	5419 (42.0)	1067 (41.7)	55,208 (42.4)	49,145 (42.4)	2371 (42.8)	3102 (42.2)	590 (41.1)
70–79	59,127 (25.6)	52,037 (25.3)	2627 (26.8)	3689 (28.6)	774 (30.3)	32,361 (24.8)	28,444 (24.5)	1447 (26.1)	2041 (27.7)	429 (29.9)
80+	18,200 (7.9)	15,954 (7.8)	878 (9.0)	1129 (8.8)	239 (9.3)	9422 (7.2)	8230 (7.1)	443 (8.0)	614 (8.3)	135 (9.4)
**Cadmium Exposure Concentration (ng/m^3^)**										
Mean	23.65	25.35	13.62	7.81	5.37	23.82	25.55	13.59	7.85	5.44
Standard Deviation	16.92	16.96	9.36	5.61	2.97	16.89	16.91	9.23	5.80	3.06
**Arsenic Exposure Concentration (ng/m^3^)**										
Mean	46.95	48.79	42.05	26.32	21.92	46.89	48.73	42.11	26.46	21.92
Standard Deviation	33.05	33.24	38.06	14.70	7.62	33.35	33.57	38.00	15.19	7.62
**Lead Exposure Concentration (ng/m^3^)**										
Mean	424.46	444.91	381.91	183.96	158.72	426.80	448.14	372.22	283.66	159.23
Standard Deviation	222.50	186.07	563.33	106.10	99.95	219.95	185.60	538.31	106.29	100.16

^a^ Cases with Gleason Score 7 were excluded from analysis as they represent an intermediate score.

**Table 2:** Adjusted and unadjusted odds ratios and 95% confidence intervals for aggressive prostate cancer for cases from the 2010–2014 Surveillance, Epidemiology and End Results (SEER) database for cadmium, arsenic, and lead each as the exposure variable comparing the 80th percentile of exposure vs. the 20th percentile in counties with different degrees of urbanization.

Measure of Aggressiveness		Metastatic vs. Not OR (95% CI)					High vs. Low Gleason Grade OR (95% CI)				
County Type		All	RUCC 1	RUCC 2	RUCC 3	RUCC 4	All	RUCC 1	RUCC 2	RUCC 3	RUCC 4
**Cadmium OR** **(95% CI)**	Unadjusted	1.01 (1.00, 1.03)	1.04 (1.01, 1.06)	1.28 (1.17, 1.40)	0.98 (0.92, 1.05)	0.88 (0.68, 1.15)	0.99 (0.98, 1.00)	1.00 (0.98, 1.02)	1.38 (1.22, 1.55)	0.96 (0.90, 1.01)	0.89 (0.76, 1.03)
	Adjusted	0.98 (0.98, 0.99)	1.01 (0.99, 1.04)	1.26 (1.14, 1.39)	0.97 (0.90, 1.05)	0.85 (0.63, 1.14)	0.99 (0.97, 1.00)	0.98 (0.96, 1.00)	1.41 (1.27, 1.57)	1.00 (0.93, 1.08)	0.94 (0.76, 1.17)
**Arsenic OR** **(95% CI)**	Unadjusted	1.04 (1.02, 1.05)	1.07 (1.05, 1.09)	1.14 (1.09, 1.18)	0.98 (0.92, 1.05)	0.86 (0.64, 1.17)	0.99 (0.97, 1.00)	1.00 (0.99, 1.02)	1.09 (1.02, 1.16)	0.94 (0.88, 0.99)	0.84 (0.68, 1.03)
	Adjusted	1.04 (1.02, 1.06)	0.97 (0.95, 0.99)	1.13 (1.08, 1.18)	1.00 (0.92, 1.08)	0.86 (0.49, 1.53)	1.00 (0.98, 1.01)	1.00 (0.97, 1.02)	1.12 (1.06, 1.18)	0.98 (0.91, 1.05)	0.78 (0.50, 1.21)
**Lead OR** **(95% CI)**	Unadjusted	1.01 (0.99, 1.03)	1.06 (1.02, 1.09)	1.00 (0.96, 1.03)	0.97 (0.91, 1.04)	0.87 (0.73, 1.03)	1.00 (0.99, 1.02)	1.02 (0.99, 1.04)	1.03 (1.00, 1.07)	0.99 (0.94, 1.05)	0.93 (0.83, 1.03)
	Adjusted	1.04 (1.03, 1.06)	0.99 (0.94, 1.04)	1.00 (0.96, 1.04)	0.98 (0.91, 1.07)	0.90 (0.76, 1.07)	1.04 (1.02, 1.06)	1.04 (1.00, 1.08)	1.04 (1.01, 1.08)	1.03 (0.97, 1.10)	0.92 (0.80, 1.05)

OR: Odds Ratio; CI: Confidence Interval. Note: Adjusted odds ratios were adjusted for age at diagnosis, county-level sociodemographic index, county-level smoking prevalence, and county-level air quality index.

**Table 3:** Adjusted odds ratios and 95% confidence intervals for aggressive prostate cancer for cadmium, arsenic, and lead each as the exposure variable for cases from the 2010–2014 Surveillance, Epidemiology and End Results (SEER) database stratified by county type and race comparing the 80th percentile with the 20th percentile of exposure.

Measure of Aggressiveness	County Type	Race	Cadmium OR (95% CI)	Arsenic OR (95% CI)	Lead OR (95% CI)
Metastatic vs. Not	All	All	0.98 (0.98, 0.99)	1.04 (1.02, 1.06)	1.04 (1.03, 1.06)
		White	1.01 (0.99, 1.03)	1.03 (1.01, 1.06)	1.01 (0.98, 1.03)
		Black	1.01 (0.97, 1.05)	0.97 (0.93, 1.01)	1.06 (1.00, 1.12)
	RUCC 1	All	1.01 (0.99, 1.04)	0.97 (0.95, 0.99)	0.99 (0.94, 1.04)
		White	1.06 (1.03, 1.08)	0.94 (0.91, 0.97)	1.03 (0.97, 1.09)
		Black	1.02 (0.96, 1.07)	0.96 (0.92, 1.01)	1.05 (0.98, 1.14)
	RUCC 2	All	1.26 (1.14, 1.39)	1.13 (1.08, 1.18)	1.00 (0.96, 1.04)
		White	1.35 (1.2, 1.52)	1.17 (1.10, 1.23)	1.00 (0.96, 1.05)
		Black	1.05 (0.7, 1.56)	0.98 (0.75, 1.27)	1.03 (0.80, 1.31)
	RUCC 3	All	0.97 (0.90, 1.05)	1.00 (0.92, 1.08)	0.98 (0.91, 1.07)
		White	1.00 (0.91, 1.10)	1.04 (0.95, 1.15)	0.95 (0.86, 1.04)
		Black	0.87 (0.70, 1.07)	0.89 (0.75, 1.06)	1.22 (0.99, 1.50)
	RUCC 4	All	0.85 (0.63, 1.14)	0.86 (0.49, 1.53)	0.90 (0.76, 1.07)
		White	0.89 (0.66, 1.21)	0.88 (0.49, 1.60)	0.90 (0.73, 1.11)
		Black	0.49 (0.04, 5.32)	2.47 (0.09, 70.72)	0.77 (0.34, 1.72)
High vs. Low Gleason Grade	All	All	0.99 (0.97, 1.00)	1.00 (0.98, 1.01)	1.04 (1.02, 1.06)
		White	0.98 (0.97, 1.00)	0.99 (0.97, 1.01)	1.04 (1.02, 1.06)
		Black	0.97 (0.93, 1.02)	0.95 (0.92, 0.99)	1.02 (0.96, 1.09)
	RUCC 1	All	0.98 (0.96, 1.00)	1.00 (0.97, 1.02)	1.04 (1.00, 1.08)
		White	0.98 (0.95, 1.00)	1.00 (0.97, 1.03)	1.05 (1.01, 1.10)
		Black	0.97 (0.92, 1.03)	0.95 (0.91, 0.99)	0.96 (0.89, 1.04)
	RUCC 2	All	1.41 (1.27, 1.57)	1.12 (1.06, 1.18)	1.04 (1.01, 1.08)
		White	1.36 (1.22, 1.51)	1.01 (0.96, 1.06)	1.05 (1.01, 1.09)
		Black	1.52 (1.05, 2.20)	1.24 (0.96, 1.60)	1.17 (0.99, 1.39)
	RUCC 3	All	1.00 (0.93, 1.08)	0.98 (0.91, 1.05)	1.03 (0.97, 1.10)
		White	0.96 (0.88, 1.05)	0.94 (0.86, 1.03)	1.02 (0.95, 1.10)
		Black	1.28 (1.10, 1.50)	1.06 (0.93, 1.20)	1.12 (0.94, 1.34)
	RUCC 4	All	0.94 (0.76, 1.17)	0.78 (0.50, 1.21)	0.92 (0.80, 1.05)
		White	0.94 (0.76, 1.17)	0.74 (0.47, 1.17)	0.93 (0.80, 1.08)
		Black	1.26 (0.16, 9.83)	1.30 (0.04, 37.91)	0.82 (0.53, 1.27)

OR: Odds Ratio; CI: Confidence Interval. Note: Adjusted odds ratios were adjusted for age at diagnosis, county-level sociodemographic index, county-level smoking prevalence, and county-level air quality index.

**Table 4:** Adjusted odds ratios and 95% confidence intervals for cases from the 2010–2014 Surveillance, Epidemiology and End Results (SEER) database for aggressive prostate cancer for cadmium, arsenic, and lead each as the exposure variable stratified by county type and age group comparing the 80th percentile with the 20th percentile of exposure.

Measure of Aggressiveness	County Type	Age	Cadmium OR (95% CI)	Arsenic OR (95% CI)	Lead OR (95% CI)
Metastatic vs. Not	All	All	0.98 (0.98, 0.99)	1.04 (1.02, 1.06)	1.04 (1.03, 1.06)
		≤60	1.05 (1.02, 1.08)	1.03 (1.00, 1.06)	1.07 (1.03, 1.10)
		61–70	1.02 (1.00, 1.05)	1.01 (0.98, 1.03)	1.04 (1.01, 1.07)
		71+	0.99 (0.97, 1.01)	1.03 (1.00, 1.06)	0.99 (0.96, 1.03)
	RUCC 1	All	1.01 (0.99, 1.04)	0.97 (0.95, 0.99)	0.99 (0.94, 1.04)
		≤60	1.07 (1.03, 1.12)	1.03 (0.98, 1.07)	1.14 (1.06, 1.23)
		61–70	1.03 (0.99, 1.08)	0.97 (0.94, 1.01)	1.09 (1.03, 1.15)
		71+	0.99 (0.96, 1.03)	0.95 (0.92, 0.98)	1.06 (1.01, 1.11)
	RUCC 2	All	1.26 (1.14, 1.39)	1.13 (1.08, 1.18)	1.00 (0.96, 1.04)
		≤60	1.25 (0.98, 1.59)	1.16 (1.03, 1.30)	1.06 (0.97, 1.14)
		61–70	1.33 (1.12, 1.57)	1.16 (1.02, 1.26)	0.99 (0.91, 1.06)
		71+	1.30 (1.02, 1.64)	1.12 (1.01, 1.24)	0.99 (0.92, 1.06)
	RUCC 3	All	0.97 (0.90, 1.05)	1.00 (0.92, 1.08)	0.98 (0.91, 1.07)
		≤60	0.84 (0.69, 1.01)	0.93 (0.78, 1.11)	1.09 (0.88, 1.36)
		61–70	1.01 (0.88, 1.16)	1.03 (0.90, 1.18)	0.97 (0.85, 1.11)
		71+	0.98 (0.87, 1.10)	0.97 (0.85, 1.11)	1.02 (0.85, 1.22)
	RUCC 4	All	0.85 (0.63, 1.14)	0.86 (0.49, 1.53)	0.90 (0.76, 1.07)
		≤60	1.03 (0.53, 2.08)	0.99 (0.29, 3.34)	0.94 (0.61, 1.45)
		61–70	0.87 (0.56, 1.37)	0.31 (0.09, 1.05)	0.89 (0.64, 1.25)
		71+	0.91 (0.68, 1.21)	1.31 (0.76, 2.26)	0.91 (0.73, 1.15)
High vs. Low Gleason Grade	All	All	0.99 (0.97, 1.00)	1.00 (0.98, 1.01)	1.04 (1.02, 1.06)
		≤60	1.01 (0.98, 1.04)	1.03 (0.99, 1.06)	1.06 (1.02, 1.09)
		61–70	1.00 (0.98, 1.01)	0.99 (0.96, 1.01)	1.03 (1.00, 1.05)
		71+	0.97 (0.95, 0.99)	1.00 (0.98, 1.03)	1.04 (1.02, 1.07)
	RUCC 1	All	0.98 (0.96, 1.00)	1.00 (0.97, 1.02)	1.04 (1.00, 1.08)
		≤60	1.01 (0.97, 1.06)	1.03 (0.98, 1.08)	1.11 (1.02, 1.20)
		61–70	1.00 (0.97, 1.03)	0.99 (0.95, 1.02)	1.02 (0.96, 1.08)
		71+	0.96 (0.93, 0.99)	1.01 (0.97, 1.05)	1.05 (0.99, 1.12)
	RUCC 2	All	1.41 (1.27, 1.57)	1.12 (1.06, 1.18)	1.04 (1.01, 1.08)
		≤60	1.46 (1.19, 1.80)	1.17 (1.05, 1.29)	1.06 (0.99, 1.13)
		61–70	1.33 (1.17, 1.51)	1.10 (1.04, 1.17)	1.02 (0.97, 1.08)
		71+	1.49 (1.21, 1.84)	1.12 (1.01, 1.23)	1.06 (0.99, 1.12)
	RUCC 3	All	1.00 (0.93, 1.08)	0.98 (0.91, 1.05)	1.03 (0.97, 1.10)
		≤60	1.09 (0.96, 1.24)	1.04 (0.92, 1.19)	1.06 (0.91, 1.24)
		61–70	1.11 (1.01, 1.21)	1.02 (0.92, 1.13)	1.06 (0.97, 1.16)
		71+	0.96 (0.85, 1.07)	0.91 (0.82, 1.02)	0.99 (0.89, 1.11)
	RUCC 4	All	0.94 (0.76, 1.17)	0.78 (0.50, 1.21)	0.92 (0.80, 1.05)
		≤60	1.08 (0.70, 1.68)	0.80 (0.36, 1.76)	0.98 (0.72, 1.35)
		61–70	0.83 (0.59, 1.18)	0.51 (0.24, 1.08)	0.87 (0.71, 1.08)
		71+	1.01 (0.76, 1.34)	1.06 (0.58, 1.93)	1.01 (0.83, 1.22)

OR: Odds Ratio; CI: Confidence Interval. Note: Adjusted odds ratios were adjusted for age at diagnosis, county-level sociodemographic index, county-level smoking prevalence, and county-level air quality index.

## Data Availability

Data were obtained from SEER registries and are available with the permission from SEER.
